# Using theory-based messages to motivate U.S. pregnant women to prevent cytomegalovirus infection: results from formative research

**DOI:** 10.1186/s12905-017-0482-z

**Published:** 2017-12-14

**Authors:** Denise M. Levis, Christina L. Hillard, Simani M. Price, Erika Reed-Gross, Erika Bonilla, Minal Amin, Jennifer D. Stowell, Rebekah Clark, Delaney Johnson, Karen Mask, Cynthia Carpentieri, Michael J. Cannon

**Affiliations:** 1 National Center on Birth Defects and Developmental Disabilities, Centers for Disease Control and Prevention (CDC), 1600 Clifton Road, Mailstop E-86, Atlanta, GA 30333 USA; 2 Carter Consulting, Inc., 2310 Parklake Drive NE, Suite 535, Atlanta, GA 30345 USA; 3 Westat, Inc., 1600 Research Blvd, Rockville, MD 20850 USA; 4 National Center for Immunization and Respiratory Diseases, Centers for Disease Control and Prevention (CDC), 1600 Clifton Road, Mailstop G-18, Atlanta, GA 30333 USA

**Keywords:** Cytomegalovirus, Congenital, Health education, Pregnant women, Fear appeals, Qualitative research

## Abstract

**Background:**

An estimated 1 in 150 infants is born each year with congenital cytomegalovirus (CMV); nearly 1 in 750 suffers permanent disabilities. Congenital CMV is the result of a pregnant woman becoming infected with CMV. Educating pregnant women about CMV is currently the best approach to prevention. Limited research is available on how to effectively communicate with women about CMV. We conducted formative research on fear appeals theory-based messages about CMV and prevention with U.S. women. Fear appeal theories suggest that message recipients will take action if they feel fear.

**Methods:**

First, we conducted in-depth interviews (*N* = 32) with women who had young children who tested positive for CMV. Second, we conducted eight focus groups (*N* = 70) in two phases and two cities (Phase 2: Atlanta, GA; Phase 3: San Diego, CA) with pregnant women and non-pregnant women who had young children. Few participants knew about CMV before the focus groups.

Participants reviewed and gave feedback on messages created around fear appeals theory-based communication concepts. The following concepts were tested in one or more of the three phases of research: CMV is severe, CMV is common, CMV is preventable, CMV preventive strategies are similar to other behavior changes women make during pregnancy, CMV preventive strategies can be incorporated in moderation to reduce exposure, and CMV is severe but preventable.

**Results:**

Participants recommended communicating that CMV is common by using prevalence ratios (e.g., 1 in 150) or comparing CMV to other well-known disabilities. To convey the severity of CMV, participants preferred stories about CMV along with prevention strategies. Participants also welcomed prevention strategies when it included a message about risk reduction. In general, participants said messages were motivating, even if they felt that it could be difficult to make certain behavior changes.

**Conclusions:**

Findings from this research can contribute to future efforts to educate pregnant women about CMV, especially regarding use of fear appeals-based messages. Pregnant women may face certain challenges to practicing prevention strategies but, overall, are motivated make changes to increase their chances of having a healthy baby.

## Background

Congenital cytomegalovirus (CMV) is the most common congenital infection in the U.S., with approximately 1 in 150 infants born with CMV each year. Nearly 1 in 750 will suffer permanent disabilities, such as hearing and vision loss, intellectual disability, psychomotor delays, and speech and language impairments [1–3].

Most healthy children and adults who contract CMV experience no symptoms and do not know that they have been infected.

There are two ways a congenital CMV infection can occur. The first is if a woman catches a primary CMV infection right before or during pregnancy [4]. The second way is if a pregnant woman experiences a reactivation of CMV or contracts a different strain [5, 6]. Young children (i.e., toddlers) are a notable source of maternal infection because they shed CMV in their urine and saliva at higher levels than people in other age groups [7, 8]. In this scenario, maternal infection can occur if a pregnant woman is exposed, via her eyes, nose, or mouth, to an infected child’s saliva or urine [9, 10].

There is no vaccine to prevent CMV infection [11–13] and treatment options for an infected fetus are limited [14–16]. U.S.-based professional associations for health care providers do not routinely recommend that providers counsel pregnant women about CMV, though professional groups in several other countries, including Australia and France, do [17]. Currently, the most promising approach to reducing risk of CMV infection is to educate pregnant women about CMV and provide them with strategies for prevention [18]. A number of researchers are trying to understand how education and behavioral interventions can reduce CMV infections [19–21].

Surveys estimate that only 14%–16% of U.S. women have heard of CMV, and even fewer are familiar with potential outcomes of congenital infection, how it is transmitted, or how to prevent transmission [22, 23]. In the United States, where we conducted our formative research, there have been no national campaigns, though there are several active advocacy groups that work in their localities to reach policymakers and pregnant women with information about CMV. Recently, for example, a new state law in Utah directed its state health department to increase awareness of CMV among pregnant women through educational programs [24]. CMV advocacy groups plan to leverage the Utah law as momentum for introducing similar legislation in other states.

To our knowledge, our research is the first to examine women’s feedback on CMV messages and prevention strategies. Theory-based messages and an empirical evidence base, including formative research, are critical for any health communication effort, and especially for CMV, where little is known about how women respond to information about CMV, the feasibility of adopting behavior changes to reduce risk of transmission, and what might motivate women to adopt new behaviors or hinder them from changing current behaviors.

### Using fear appeals theories to inform message development

With no previous research available on CMV messaging, we used constructs from fear appeals theories, such as protection motivation theory [25] and extended parallel process model (EPPM) [26], to guide initial concept and message development. Incorporation of empirically-tested theory into messaging can better ensure that messages about CMV resonate with women and persuade them to adopt prevention strategies [27].

Fear appeal theories posit that if message recipients feel fear, they will be motivated to take action. Ideally, the message recipient will take action by following recommended behaviors or strategies for protection. Decades of research suggest that messages with strong fear appeals increase chances of improving attitudes and intentions and increase the likelihood of behavior change [28]. Messages can backfire if they induce too much fear when the recipient elects to respond defensively by managing that fear [29]. Conversely, as illustrated in a study of the persuasive effects of fear appeals in genital warts educational messages, messages can fail if they do too little to stimulate a person’s perception of threat [30].

EPPM is the most current and frequently used fear appeal theory. It integrates constructs from several other fear appeals theories, positing that individuals assess messages in layers, firstly for its threat and secondly for the action needed to reduce the threat [26]. The threat component of a message conveys the degree of harm (i.e., severity) caused by a threat and/or the likelihood (i.e., susceptibility) of the threat. A meta-analysis studying the effects of fear appeals used in public health messaging found that perceived susceptibility and perceived severity reliably influence attitudes, intentions, and behaviors, though effects are often small [28].

Perceived efficacy is the “action” construct, which includes perceived self-efficacy and perceived response efficacy [28]. Messages that integrate efficacy aim to make recipients to feel that they are able (i.e., have self-efficacy) to protect themselves from the threat and/or that the suggested protective actions work (i.e., have response efficacy). The fear appeals meta-analysis found that high-efficacy messages greatly increase the likelihood of behavior change [28]. More specifically, the study of genital warts educational messages found that study participants responded best to prevention behaviors that were detailed and doable [30].

We began Phase 1 of our formative research using three theory-based communication concepts to frame messages about CMV: 1) CMV is serious, 2) CMV is common, and 3) CMV is preventable. Across all concepts, we identified or developed a diverse group of messages that illustrated the concepts to different degrees. CMV messages either derived from language on the CDC CMV website [31] or were developed by the project team’s subject matter experts. Participants had the opportunity to discuss in detail, compare, and choose which messages they preferred best and least. The first two concepts aim to convey that CMV is a relevant and severe threat, aiming to influence perceived threat. We used messaging tactics such as numeracy (e.g., 1 in 150 babies are born with CMV), narrative (e.g., use of a personal story), and comparison (e.g., CMV causes as much disability in children as Down syndrome, fetal alcohol syndrome, or spina bifida) as we developed specific messages. For our third concept, we developed messages with information about prevention and action in an effort to bolster message recipients’ perceived self-efficacy. In addition, we used phrasing like “studies show” in a few messages to stimulate participants’ perceived response efficacy.

## Methods

### Background

We conducted three phases of formative research between 2011 and 2013. Research approval was obtained through Institutional Review Boards at the National Center on Birth Defects and Developmental Disabilities at Centers for Disease Control and Prevention (Phase 1) and Westat, Inc. (Phases 2 and 3). All participants gave informed consent and were compensated for time and travel.

### Phase 1 data collection

In Phase 1, we asked participants (*N* = 32) enrolled in a longitudinal CMV study in Atlanta, GA, to complete one-on-one interviews at the end of the 12-week study. Recruitment methods and information about the longitudinal study are described elsewhere [8].

All participants were women and had at least some knowledge of CMV due to their participation in the longitudinal study. All had at least one child < 3 years old who tested positive for CMV and most knew their own CMV serostatus. English proficiency was a requirement during participant recruitment, but a small number of interviews (*n* = 4) were conducted in Gujarati because participants felt more comfortable discussing CMV in their native language.

Four trained study personnel, including one fluent in Gujarati and English, conducted the interviews in teams of two. During the interview, one person conducted the interview and the other took notes. Each interview was audio recorded and lasted 40 min on average.

During each interview, messages (Table 1) were presented in large, plain font on three pieces of paper, divided by the three communication concepts. Participants reviewed and gave feedback on the messages, one concept at a time. The interviewer probed for reactions, asked for opinions about the concordance of the message with the concept, asked about participants’ favorite and least favorite message per group, and requested suggestions for improvement. Feedback was also collected on the prevention strategies listed in Message #15. Discussion focused on participants’ comprehension of strategies, their motivation to adopt the strategies, feasibility of doing them, and anticipated barriers.

**Table 1:** CMV messages tested during Phase 1, by communication concept

Communication Concept 1: CMV is severe. 1. Congenital CMV is a very serious condition. 2. Some babies born with congenital CMV develop disabilities such as hearing loss, vision loss, or mental disability. 3. Congenital CMV is one of the most common causes of birth defects. 4. Emma is not a typical three-year-old. She rarely rolls over and cannot sit up on her own. She makes lots of noise, but has yet to speak a word. She does not drink from a sippy cup or feed herself - most of her food is provided through a feeding tube. She has multiple seizures each day. Developmentally, she is five months old. These issues, along with many others, are the result of Emma’s congenital CMV.	Communication Concept 2: CMV is common. 5. CMV is the most common congenital infection. 6. Each year, 30,000 babies are born with CMV. 7. 1 in 150 babies are born with congenital CMV infection. 8. 1 in 750 babies has a disability due to congenital CMV. 9. Each year, 5500 babies develop a disability due to congenital CMV. 10. Congenital CMV causes as much disability in children as Down syndrome, fetal alcohol syndrome, or spina bifida.	Communication Concept 3: CMV is preventable. 11. Congenital CMV is preventable. 12. You can protect your baby from congenital CMV. 13. A pregnant woman can prevent transmission of CMV by washing hands often and trying to avoid getting a young child’s urine or saliva in her eyes, nose, or mouth. 14. Studies show that pregnant women can prevent CMV infection by following a few basic prevention guidelines. 15. Avoiding contact with urine or saliva—especially from preschool children—can lower your chance of getting CMV and passing it to your unborn baby. Here are a few simple steps to avoid getting urine and saliva in your eyes, nose, or mouth: • Wash your hands often with soap and water, especially after - changing diapers, - feeding a child, - wiping a child’s nose or mouth, - handling children’s toys. If water is not available, use an alcohol-based hand sanitizer. • Do not share food, drink, or utensils with young children. • Do not put a child’s pacifier in your mouth. • Clean toys, countertops, and other surfaces that come into contact with children’s urine or saliva with soap and water or a disinfectant. • Avoid contact with saliva when kissing a young child.

### Phase 1 data analysis

Professional transcriptionists produced verbatim transcripts of audio recordings. The Gujarati transcripts were transcribed and translated by a study team member, different from the interviewer, who was fluent in Gujarati and English.

Two members of the study team coded the transcripts. Codebook development was initially guided by the messages. We began with one category for each individual message (Table 1) and each prevention strategy (Table 1, #15). Repeated review of the transcripts led to subcategories for each message: positive opinions, negative opinions, and suggestions for edits. To test inter-coder reliability, the coding team piloted three rounds of coding using a small random sample (< 5) of transcripts. Coding of all transcripts commenced once inter-coder reliability reached > 80% overall. The qualitative data analysis program QSR NVivo v9.2 facilitated the analysis by providing descriptive reports for each category. The project team reviewed all final thematic categories. Disagreements were resolved through discussion and consensus.

### Phases 2 and 3 data collection

In Phase 2 we conducted four focus groups with women (*N* = 38) in San Diego, CA, and in Phase 3, we conducted four focus groups with women (*N* = 32) in Atlanta, GA. Because CMV birth prevalence is significantly higher among Black women than White women [3], we grouped Black women together in two groups at each of the two sites and did the same with White women. All other racial and ethnic groups were excluded. Recruitment firms recruited women using their existing databases. Potential participants were recruited and screened via telephone. In addition to race, screening criteria included women who were either currently pregnant or planning a pregnancy in the next 12 months and who lived with a child < 5 years old, were proficient in English, and 18–40 years of age.

One of two trained female moderators led each focus group discussion. Focus groups were audio recorded, and project team members took notes behind a two-way mirror. Discussions typically lasted 90 min.

In contrast to participants from Phase 1, who had some familiarity with CMV, we assumed that focus group participants would know little or nothing about CMV, given estimates of low national awareness. Therefore, the moderator provided a brief introduction to CMV before message testing commenced. Otherwise, the approach to message testing was similar to Phase 1. Due to time constraints, the Phase 2 moderator’s guide ncluded a shorter list of messages, some of which were revised based on feedback from Phase 1 (Table 2). Message testing in phases offers researchers the opportunity to rapidly respond to participants’ concerns and collect feedback on multiple iterations of messages [32]. By the end of Phase 2, the study team felt that feedback saturation had been reached for many of the messages. Taking advantage of feedback received during Phases 1 and 2, Phase 3 explored reactions to new concepts and messages we created (Table 3). Messages in Table 3 were developed by the project team’s subject matter experts.

**Table 2:** CMV messages tested during Phase 2, by communication concept

Communication Concept 1: CMV is severe. 1. Some babies born with congenital CMV develop disabilities such as hearing loss, vision loss, or mental disability. 2. Congenital CMV is one of the most common causes of birth defects. 3. Mark was born with congenital CMV. By nine months old he still could not crawl and did not respond to his name. We had his hearing checked and found out that he had some hearing loss in both ears. As he grew older, Mark also had some trouble walking. Despite these problems, he is now a happy eight year old boy who loves to play video games and laugh.	Communication Concept 2: CMV is common. 4. 1 in 750 babies has a disability due to congenital CMV. 5. Each year 5500 babies develop disabilities due to CMV. 6. Congenital CMV causes as much disability in children as Down syndrome, fetal alcohol syndrome, or spina bifida.	Communication Concept 3: CMV is preventable. 7. Congenital CMV is preventable. 8. A pregnant woman can prevent transmission of CMV by washing hands often and trying to avoid getting a young child’s urine or saliva in her eyes, nose, or mouth. 9. Prevent CMV infection when you are pregnant by: • Not putting things in your mouth that have just been in a child’s mouth. For example: food, cups or silverware, toothbrush, pacifier. • Trying to avoid getting saliva in your mouth when kissing a child. You can do this by kissing on the cheek or forehead instead of the lips. • Washing your hands after touching a child’s urine or saliva.

**Table 3:** CMV messages tested during Phase 3, by communication concept

Communication Concept 1: CMV preventive strategies are similar to other behavior changes women make during pregnancy. 1. Women make lots of changes while they are pregnant, like not eating certain types of fish, not drinking alcohol and caffeine and not smoking. Adding a few more changes into your routine can help keep your unborn baby safe from CMV. These changes might include washing your hands often and trying to avoid getting a young child’s urine and saliva in your eyes, nose, or mouth. Keep in mind, these are changes you only need to make during pregnancy.	Communication Concept 2: Preventive strategies can be incorporated in moderation to reduce exposure. 2. Congenital CMV is one of the most common causes of birth defects, but there are ways you can reduce the risk to your unborn baby. Although it may be hard to avoid all possible exposures to CMV, by making a few recommended changes while you are pregnant, you can help protect your unborn baby from infection. These include: • Avoid putting things in your mouth that have just been in a child’s mouth. When possible, try not to share food, cups, or silverware with your child or put their pacifier in your mouth. • Avoid getting saliva in your mouth when kissing a child. You can do this by trying to give more kisses on the cheek or forehead instead of the lips. • Clean your hands after touching a child’s urine or saliva. For example, try to make a habit of cleaning your hands after changing a diaper, feeding a child, or wiping a child’s nose or mouth.	Communication Concept 3: CMV is severe but preventable. 3. My son Mark was born with congenital CMV. By nine months old he still could not crawl and did not respond to his name. I had his hearing checked and found out that he was deaf in both ears. As he grew older, Mark also had some trouble walking. I learned from his doctors that these issues were caused because I was exposed to CMV while I was pregnant with him. I wish I had known about the simple things I could have done to keep Mark from getting this virus. All moms should know that there are things they can do to protect their unborn babies from being exposed to congenital CMV. You can prevent CMV infection when you are pregnant by: • Not putting things in your mouth that have just been in a child’s mouth. For example: • Food • Cups or silverware • Toothbrush • Pacifier • Avoid getting saliva in your mouth when kissing a child. You can do this by kissing on the cheek or forehead instead of the lips. • Washing your hands after touching a child’s urine or saliva. For example, after: • Changing diapers • Feeding a child • Wiping a child’s nose or mouth.

### Phases 2 and 3 data analysis

Professionals transcribed each focus group. Initial categories for the codebook were formed using the moderator’s guide as well as the concepts and messages tested. Additional review yielded other recurring themes and subcodes. Three members of the project team read each transcript and used a team-based approach to coding reliability; they shared the codebook and coding data with one another throughout the process and resolved disagreements through discussion and consensus. QSR NVivo v9.0 facilitated the analysis by providing descriptive reports for each category. The project team reviewed all final thematic categories, and disagreements were resolved through discussion and consensus.

## Results

### Phase 1 results

Phase 1 participants were primarily Non-Hispanic White and included a small segment of Asian (Indian) women (Table 4). Most participants had a college degree or higher.

**Table 4:** Demographic characteristics across three phases of formative research

	Phase 1(*N* = 32)	Phase 2(*N* = 38)	Phase 3(*N* = 32)
Characteristic			
Age			
18–25	1 (3%)	10 (26%)	2 (6%)
26–30	4 (13%)	14 (37%)	8 (25%)
31–35	15 (47%)	7 (18%)	10 (31%)
36–40	10 (31%)	7 (18%)	12 (38%)
41–45	2 (6%)	--^a^	--^a^
Race/Ethnicity			
Asian-American	9 (28%)	--^a^	--^a^
Black or African-American	2 (6%)	20 (53%)	16 (50%)
White or Caucasian	18 (56%)	18 (47%)	16 (50%)
Hispanic-Latina	3 (9%)	--^a^	--^a^
Education Level			
High school diploma, GED, or <high school	3 (9%)	3 (8%)	3 (9%)
Technical college, Associate degree, or some college	2 (6%)	19 (50%)	7 (22%)
College degree or more	27 (84%)	16 (42%)	22 (69%)
Pregnancy Status			
Planning	--^a^	21 (55%)	26 (81%)
Pregnant	1 (3%)	17 (45%)	6 (19%)

^a^In corresponding phase of research, women in this category were excluded, or category of data was not collected

### Phase 1 message testing

#### CMV is severe

After reviewing messages about the severity of CMV (Table 1, #2-#4), some suggested combining the messages about sequelae (#2) and birth defects (#3) for greater impact. About two-thirds of participants said the story about a child with CMV (#4) was attention getting; others, however, felt that the story was scary, and that prevented them from wanting to learn more about CMV. Some felt that the story featured a rare case and, thus, it did not increase their feelings of susceptibility towards CMV. For instance, one participant said, “This [story] seems a little too — I mean I understand it’s illustrating, but it’s almost too hit-you- over-the-head. I think it overshoots its mark.”

#### CMV is common

Participants preferred prevalence ratio numeracy messages (Table 1, #7 and #8) over raw number messages (#6 and #9) because the ratios offered context for understanding the issue. Additionally, as one participant said, “Um, I mean these two: the 1 in 150 and 1 in 750. I like those better just because oh, you know, 150 babies that’s not that many so that hits home more than these bigger numbers.” Message 10, which compared the number of cases of CMV to other well-known childhood disabilities, was favored by most participants. Message 5 was poorly received because it used the term “congenital” without providing a definition.

#### CMV is preventable

Brief messages about prevention (Table 1; #11, #12, and #14) received negative feedback, while messages containing specific prevention strategies (#13 and #15) were favorably reviewed.

Despite positive feedback on the overall concept of message 15 (Table 1), many participants cited concerns with the message’s prevention strategies. Most participants read and understood an implied message, the need for constant vigilance against transmission, which participants said would take too much time and was unrealistic. For instance, most participants felt it would be difficult to avoid all exposure to a young child’s saliva because it is so prevalent with young children who are teething, affectionate, mouthing objects, and wanting to share food and drink. It would also be difficult to find time to wash hands after handling toys or even regularly washing toys. Hand washing after wiping a child’s nose or mouth would be hard to remember to do each time and could potentially be done so often it would leave a person’s hands raw. Participants also cited challenges with changing their habits or routines (e.g., kissing on the mouth). Regarding avoiding kissing on the mouth, one participant said, “If I knew it was going to make my unborn child potentially sick, I would just avoid it. But it would be sad that I would have to avoid kissing my child on the lips.”

Many participants also said they did not plan to become pregnant again and, therefore, were not motivated to prevent exposure to CMV. However, they thought the prevention strategies were important and planned to share them with friends and relatives who were pregnant or planning a pregnancy. Another barrier unique to this group was their knowledge of their own CMV serostatus, which they received along with their child’s as part of the CMV study. Most were seropositive like their child(ren) (29/32). They felt that the prevention strategies were irrelevant since they were already infected.

In general, participants felt that having a healthy pregnancy was an important motivator for behavior change. Even women with no plans to become pregnant agreed that, in theory, another pregnancy would motivate them to adopt the necessary behavior changes. Participants also cited facilitators like having ready access to appropriate cleaning products (e.g., hand sanitizer) to address concerns about finding time to wash hands and knowing about CMV and how to prevent it.

### Phase 1 message decisions

In anticipation of further phases of testing, some messages, especially those with prevention strategies (Table 1, #13 and #15), were revised based on Phase 1 feedback. Scientific information that emerged from the longitudinal CMV study [8] also contributed to revisions to the prevention strategies in Phase 2 and 3 prevention messages. Time constraints related to collecting feedback on messages during focus groups prevented us from retesting all Phase 1 messages in Phase 2, so not all were chosen for additional testing. Certain messages that received mostly positive feedback were selected (#2, #3, #8, #10, and #13), and two messages that received mostly negative feedback were selected (#9 and #11).

### Phases 2 and 3 overall results

Pregnant women and women planning a pregnancy each accounted for about 50% of participants in Phase 2. The majority of participants in Phase 3 were planning a pregnancy, and only about 20% of participants were pregnant (Table 4). All women in Phases 2 and 3 lived with a child <5 years old. Most (91%) participants in Phases 2 and 3 had either some college education or a college degree.

**Fig. 1:**
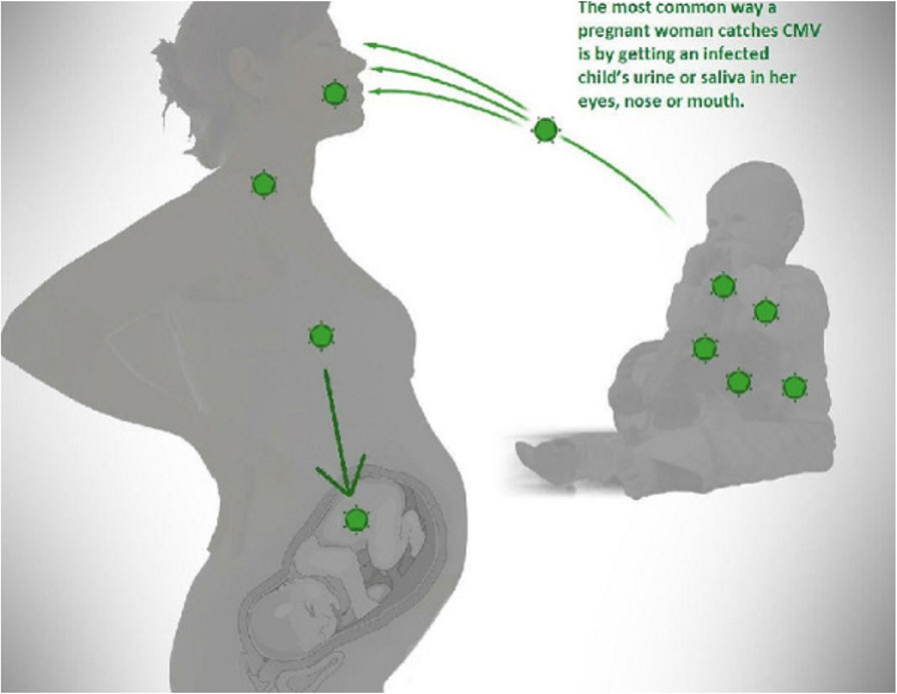
CMV Infection Transmission Illustration. Child-to-mother transmission graphic shown to women during focus groups (Phases 2 and 3)

During the focus group discussions, the moderator first polled participants about their knowledge of CMV. Nearly all participants (*n* = 68 [99%]; 2 abstained) reported no prior knowledge of CMV. Next, the moderator provided each group with a brief description of CMV and shared a diagram of child-to-mother transmission (Fig. 1). Participants’ most common reaction was to raise additional questions about CMV (Table 5); they also expressed fear, apathy, and anger. Anger and fear were expressed among all women, but especially among those who were planning pregnancies. Participants who expressed fear said the term “cytomegalovirus” sounded scary and unfamiliar. Those who expressed anger questioned why so few knew about CMV and were frustrated that their health care providers had not told them about it. Apathy was found predominantly among pregnant women in Phase 2 because they felt that they already had enough to worry about during their pregnancy.

**Table 5:** Number of participants’ frequently asked questions about CMV during Phase 2 and 3 focus groups

How is CMV spread?	42
How does CMV work/ cause disabilities?	25
Can my toddler, spouse, or I get tested for CMV infection?	22
Do doctors check newborns for CMV-related effects/ symptoms?	13
Is there a vaccine (or other form of prevention)?	9
How do I know my child is infected?	9
What are the statistics (e.g., prevalence)?	9
Is there a treatment or cure?	7
Is CMV something new?	7
How old are the young children who typically spread CMV?	7
Other	15
TOTAL	165

### Phase 2 message testing

*CMV is severe*. The majority of the feedback focused on the story about the child with CMV (Table 2, #3). Participants said the story sent a mixed message about severity, and they could not discern if the child with CMV outgrew his disabilities. Reiterating the apathy expressed earlier in the discussion, many pregnant participants felt overburdened by the messages within this concept, feeling that CMV was one more thing to worry about during pregnancy. Many participants also expressed disbelief and distrust in the accuracy of the messages, saying, for example, “How is it one of the most common causes of birth defects, and nobody knows about this?”

#### CMV is common

Participants in all but one group reported that the prevalence ratio (Table 2, #4) increased their perception of risk. Some wanted additional information to help them understand CMV prevalence (e.g., was the prevalence applicable to everyone in the United States or San Diego only? Were there differences among racial or ethnic groups?). The comparison of CMV to other birth defects (#6) was rated as most compelling. However, it also confused some participants, who had difficulty discerning if CMV was as *severe* as the other disabilities or as *common*.

#### CMV is preventable

Messages in this group received mostly positive feedback and were described as “optimistic” (Table 2). A majority of participants found the messages easy to understand and “not a scare tactic,” although some wanted to know how “young child” was defined (i.e., what is the age range during which a child would likely spread CMV?).

Some participants had concerns about following the recommendation to avoid saliva when kissing a child (#9). Participants who shared affection by kissing their child on the lips expressed reluctance to adjusting the behavior. Participants also mentioned two habits related to food sharing that they said would be difficult to give up: sharing drinks with a child and allowing a child to taste food from a mother’s plate. Fewer concerns were mentioned with washing hands after touching a child’s saliva or urine, although some participants said washing hands after feeding a child or after wiping a child’s nose or mouth was not part of their routine, so it would be difficult to remember to do.

Facilitators to following the behaviors included increased knowledge of CMV prevalence; some described being motivated if they knew that CMV was more common—for example, as prevalent as a virus like influenza. Other motivators included being pregnant (“The minute I find out I’m pregnant? I would probably stop the next day from letting my kid eat off my fork or drink out of my cup.”), knowing about the severity of CMV, and thinking about how the advice supports good hygiene in general.

### Phase 2 message decisions

The team determined that feedback saturation had been reached for most of the messages, but felt it was important to revise and retest the personal story about CMV (Table 2, #3) because Phase 2 participants shared many concerns about it. We also created two new messages to test in Phase 3 that were inspired by participant feedback.

### Phase 3 message testing

*Preventive behaviors are similar to other behavior changes.* This message (Table 3, #1) was designed to address the apathy expressed in Phase 2 and participants’ reticence to attend to another risk during pregnancy. But some participants still felt overwhelmed and stressed by the suggestion of the prevention measures. To illustrate, one participant said, “I feel like they’re going ‘you already can’t do all of the stuff you used to really like to do. And then there’s even more stuff you need to be careful of, even more things that can go wrong.’ That’s the way the message comes across to me.” Participants said that this message could be motivating if it included health effects of CMV. Participants in a few groups also described being confused about the “only during pregnancy” aspect of the message, which made them think that a woman cannot get infected with CMV if she is not pregnant.

#### Preventive behaviors can be incorporated in moderation to reduce exposures

Risk reduction was incorporated into this message (#2) in response to participants’ concerns about the prevention strategies being unrealistic and how difficult it would be to change so many habits. Many Phase 3 participants found this to be a realistic and approachable message, remarking that the person who wrote it had spent time around children. Participants also understood that risk reduction could be an effective strategy; for example, “What they’re saying is that you can’t do it 100%. I feel like it raises awareness and it lets people know to make sure of these things and change this behavior. It’s not possible to avoid all of them, but even if you take these steps you can better you chances of not getting it.”

#### Congenital CMV is severe but preventable

Participants expressed about an equal number of positive and negative opinions about the revised story (#3). Those who responded positively said they liked that it included a real-life experience with CMV and that it invoked both a visual and emotional response. One participant said, “It gives you a visual. When you read out not crawling or hearing I was picturing her at the doctor’s office having to hear that and the baby not crawling. That’s devastating to me.” Conversely, others felt that the personal story unnecessarily blamed the mother for not following prevention strategies and the doctor for not educating women about CMV. A few participants objected to the story because they felt it was too emotional; these participants preferred factual information about CMV (“I don’t need the sob story, I just want facts.”). Despite the negative feedback, most participants preferred this message to the others they reviewed.

We polled participants about their likelihood to follow prevention strategies (Table 3, #3). Most said they would be likely or somewhat likely to follow the recommended strategies (Table 6). Discussion about prevention strategies yielded responses similar to those from Phase 2. Many participants found that habits like kissing a child on the lips or sharing food would be difficult to change, although knowing more about CMV prevalence and having the right cleaning supplies on hand could likely facilitate efforts to make behavior changes. Having a health care provider talk with participants about CMV would also motivate them to adopt prevention strategies.

**Table 6:** Phase 3 participants’ (*n* = 32) likelihood of following CMV prevention strategies

Guideline	Very likely to follow No.(%)	Somewhat likely to follow No.(%)	Not at all likely to follow No.(%)
Do not put things in your mouth that have just been in a child’s mouth.	15 (47%)	16 (50%)	1 (3%)
Avoid getting saliva in your mouth when kissing a child.	18 (56%)	11 (34%)	3 (9%)
Wash your hands after touching a child’s urine or saliva.^a^	29 (94%)	2 (6%)	0

^a^One participant did not vote

## Discussion

We report some of the first research on women’s feedback on theory-based messages about CMV. Our findings can help shape how CMV and prevention strategies ought to be promoted by practitioners, and it offers important insight to the U.S. Centers for Disease Control and Prevention as it continues to review and consider its promotion of CMV prevention strategies. Our findings are also instructive to others who use fear appeals theories such as EPPM in health messaging.

In Phases 2 and 3, most participants were not aware of CMV. Participants’ low awareness of CMV could have contributed to the apathy, disbelief, and fear they felt when they initially learned about CMV. In turn, according to EPPM, such reactions could cause participants to reject information about CMV [29, 30]. Most importantly, these results suggest the need to increase women’s awareness of CMV. As long as awareness remains low, our findings indicate that practitioners who promote CMV prevention ought to consider incorporating as much information about CMV as possible, including key prevention information.

We also found that, if employed effectively, stories could be an effective way to introduce CMV and prevention strategies. Through our multi-phase testing process, we learned that stories about CMV run the risk of being rejected out of disbelief or fear if the story (i.e., health effects of CMV on babies) is too extreme. In our study, it took several iterations of the story to strike a balance between conveying the severity of CMV without inducing excessive fear or skepticism. Stories are a common way to share critical information about health topics, including CMV. Practitioners who use stories to promote CMV ought to consider testing them with their target audience in order to better locate the fine line between motivation to act and rejection.

Regarding perceived susceptibility, we learned that context is important in messages about CMV. Participants preferred the “1 in 150” numeracy message (Table 1, #7) over the “30,000” numeracy message (Table 1, #6). This desire for context also could explain why the message comparing CMV to other more well-known conditions (Table 1, #10) attracted participants’ attention and received favorable reviews.

The risk reduction concept, which was developed from Phase 1 and Phase 2 findings and shown in Phase 3, also seemed to improve women’s self-efficacy and motivation to follow prevention strategies. The message made the prevention behaviors seem more doable. Some barriers to the prevention strategies persisted through Phases 2 and 3, and these are worth noting. Phase 2 participants voiced concerns about not kissing their child on the lips, which would mean changing how they shared affection with their children. Practitioners might consider pairing this message with images of mothers sharing affection with their child by cuddling or hugging, or providing alternative suggestions for kissing—such as on the forehead or cheek—to mitigate these concerns. Phase 2 and 3 participants were also concerned about the effort it would take to break certain habits, such as sharing food and drink, that they have developed. Providing alternative suggestions, such as having separate utensils for the mother and child and giving children beverages in their own cups, could address these concerns. Partnership opportunities for CMV remain relatively unexplored and are important strategy to consider as well. For example, companies that sell hand sanitizer or utensils and cups for children might want to collaborate and contribute samples for distribution during a coordinated communication campaign.

The majority of participants said they would likely follow the prevention strategies because having a healthy pregnancy and having a healthy baby were important motivators to them. Other research confirms that pregnancy is a key trigger for motivating new, healthy behaviors in women [33]. Gain-framed messages that highlight the benefits of having a healthy baby when following CMV prevention strategies could be beneficial to test in future studies.

We also found that some of the Phase 1 participants who were CMV positive said that they were not motivated to prevent CMV transmission. These participants were likely unaware that they could either become infected with another strain or that their inactive virus could reactivate. It is important for practitioners, therefore, to emphasize that *all* pregnant women, regardless of CMV status, should avoid exposures to CMV during pregnancy.

Some caution should be taken in interpreting our results. Findings are not generalizable to all U.S. women. Assignment by certain shared characteristics (e.g., pregnant or planning a pregnancy) creates homogeneity within the groups, aiding in group dynamics and discussion and helping the researchers to identify major differences between groups [34]. The characteristics we used were quite broad, and may explain the limited number of differences found between the groups. Most participants had more than a high school education, which likely influenced results because they may have been better able to understand the inherent complexities of messages about CMV and had fewer questions and/or information needs than women with average or below-average education. Additional research is needed to test CMV messages with women with below-average education. Additionally, fear appeals can be communicated in a variety of ways (e.g., using images, changing font size, etc.) to emphasize messages. In our study, messages were shown to participants in plain text, without any other design elements which, in a coordinated communication campaign, are central to attracting attention and increasing motivation. A follow-up study assessed two health education materials, including images and messages, promoting CMV information and prevention strategies; materials were favorably reviewed and increased women’s knowledge of CMV and motivation to follow prevention strategies [35].

## Conclusions

In summary, our research highlights the benefits of using fear appeals theories to underpin messages about CMV. It also underscores the need for increased awareness and the importance of conducting regular audience testing with pregnant women while awareness of CMV remains low. Feedback on prevention strategies also highlight some of the challenges that pregnant women may face when they try to prevent transmission, though risk reduction messages could mitigate these challenges and motivate women. Findings from this research can contribute to successful efforts to educate pregnant women about CMV with the goal of preventing transmission during pregnancy.

## Abbreviations

CMV: Congenital Cytomegalovirus
EPPM: Extended Parallel Process Model

## References

[1] Arvin AM, Fast P, Myers M, Plotkin S, Rabinovich R (2004). Vaccine development to prevent cytomegalovirus disease: report from the National Vaccine Advisory Committee. Clin Infect Dis.

[2] Dollard SC, Grosse SD, Ross DS (2007). New estimates of the prevalence of neurological and sensory sequelae and mortality associated with congenital cytomegalovirus infection. Rev Med Virol.

[3] Kenneson A, Cannon MJ (2007). Review and meta-analysis of the epidemiology of congenital cytomegalovirus (CMV) infection. Rev Med Virol.

[4] Mocarski ES, Shenk T, Pass RF, Knipe DM, Howley PM (2007). Cytomegaloviruses. Fields’ virology.

[5] Boppana SB, Rivera LB, Fowler KB, Mach M, Britt WJ (2001). Intrauterine transmission of cytomegalovirus to infants of women with preconceptional immunity. N Engl J Med.

[6] Nyholm JL, Schleiss MR (2010). Prevention of maternal cytomegalovirus infection: current status and future prospects. Int J Womens Health.

[7] Cannon MJ, Hyde TB, Schmid DS (2011). Review of cytomegalovirus shedding in bodily fluids and relevance to congenital cytomegalovirus infection. Rev Med Virol.

[8] Stowell JD, Mask K, Amin M, Clark R, Levis DM, Hendley W (2014). Cross-sectional study of cytomegalovirus shedding and immunological markers among seropositive children and their mothers. BMC Infect Dis.

[9] Pass RF, Little EA, Stagno S, Britt WJ, Alford CA (1987). Young children as a probable source of maternal and congenital cytomegalovirus infection. New Engl J Med..

[10] Adler SP (1989). Cytomegalovirus and child day care. New Engl J Med..

[11] Khanna R, Diamond DJ (2006). Human cytomegalovirus vaccine: time to look for alternative options. Trends Mol Med.

[12] Schleiss MR (2008). Cytomegalovirus vaccine development. Human Cytomegalovirus.

[13] Pass RF, Zhang C, Evans A, Simpson T, Andrews W, Huang ML (2009). Vaccine prevention of maternal cytomegalovirus infection. New Engl J Med..

[14] Nigro G, Adler SP, La Torre R, Best AM (2005). Passive immunization during pregnancy for congenital cytomegalovirus infection. New Engl J Med..

[15] Revello MG, Lazzarotto T, Guerra B, Spinillo A, Ferrazzi E, Kustermann A (2014). A randomized trial of hyperimmune globulin to prevent congenital cytomegalovirus. New Engl J Med..

[16] Kimberlin DW, Jester PM, Sánchez PJ, Ahmed A, Arav-Boger R, Michaels MG (2015). Valganciclovir for symptomatic congenital cytomegalovirus disease. New Engl J Med.

[17] Griffiths P, Heath P, Hollins Martin C, Kilby M, Sharland M. We need to talk about CMV. 2015. http://cmvaction.org.uk/sites/default/files/we_need_to_talk_about_cmv_2.pdf. Accessed 15 July 2015.

[18] Bate SL, Cannon MJ (2011). A social marketing approach to building a behavioral intervention for congenital cytomegalovirus. Health Promot Pract.

[19] Adler SP, Finney JW, Manganello AM, Best AM (1996). Prevention of child-to-mother transmission of cytomegalovirus by changing behaviors: a randomized controlled trial. Pediatr Infect Dis J.

[20] Vauloup-Fellous C, Picone O, Cordier AG, Parent-du-Châtelet I, Senat MV, Frydman R (2009). Does hygiene counseling have an impact on the rate of CMV primary infection during pregnancy?: results of a 3-year prospective study in a French hospital. J Clin Virol.

[21] Revello MG, Tibaldi C, Masuelli G, Frisinav V, Sacchiv A, Furionev M (2015). Prevention of primary cytomegalovirus infection in pregnancy. EBioMedicine.

[22] Jeon J, Victor M, Adler S, Arwady A, Demmler G, Fowler K (2006). Knowledge and awareness of congenital cytomegalovirus among women. Infect Dis Obstet Gynecol.

[23] Ross DS, Victor M, Sumartojo E, Cannon MJ (2008). Women's knowledge of congenital cytomegalovirus: results from the 2005 HealthStyles survey. J Women's Health.

[24] Cytomegalovirus Public Health Initiative. Utah Assemb. 2013;HB 81, Section 26–10-10.

[25] Rogers RW, Cacioppo J, Petty R (1983). Cognitive and physiological processes in fear appeals and attitude change: a revised theory of protection motivation. Social psychophysiology: a sourcebook.

[26] Witte K (1992). Putting the fear back into fear appeals: the extended parallel process model. Commun Monogr.

[27] Noar SM (2012). An audience–channel–message–evaluation (ACME) framework for health communication campaigns. Health Promot Pract.

[28] Witte K, Allen M (2000). A meta-analysis of fear appeals: implications for effective public health campaigns. Health Educ Behav.

[29] Witte K, Andersen PA, Guerrero LK (1998). Fear as motivator, fear as inhibitor: using the extended parallel process model to explain fear appeal successes and failures. Handbook of communication and emotion: research, theory, applications, and contexts.

[30] Witte K, Berkowitz JM, Cameron KA, McKeon JK (1998). Preventing the spread of genital warts: using fear appeals to promote self-protective behaviors. Health Educ Behav.

[31] Babies Born with CMV (Congenital CMV Infection). In: Cytomegalovirus (CMV) and Congenital CMV Infection. Centers for Disease Control and Prevention. https://www.cdc.gov/cmv/index.html.

[32] Brown KM, Lindenberger JH, Bryant CA (2008). Using pretesting to ensure your messages and materials are on strategy. Health Promot Pract.

[33] Squiers L, Mitchell B, Levis D, Lynch M, Dolina S, Margolis M (2013). Consumers’ perceptions of preconception health. Am J Health Promot.

[34] Krueger RA, Casey MA (2009). Focus groups. A practical guide for applied research, 4^th^ edition.

[35] Price SM, Bonilla E, Zador P, Levis DM, Kilgo CL, Cannon MJ (2014). Educating women about congenital cytomegalovirus: assessment of health education materials through a web-based survey. BMC Womens Health.

